# Operational tolerance research in liver transplantation: a bibliometric analysis using a new web resource

**DOI:** 10.3389/frma.2024.1368534

**Published:** 2024-03-14

**Authors:** Ángel Esteban-Gil, Juan José Martínez-García, Daniel Vidal-Correoso, Ana M. Muñoz-Morales, Pablo Ramírez, José Antonio Pons, Alberto Baroja-Mazo

**Affiliations:** ^1^Biomedical Informatic and Bioinformatic Platform, Biomedical Research Institute of Murcia (IMIB-Arrixaca), Murcia, Spain; ^2^Department of Biochemistry and Molecular Biology B and Immunology, Faculty of Medicine, University of Murcia, Murcia, Spain; ^3^Digestive and Endocrine Surgery and Transplantation of Abdominal Organs, Biomedical Research Institute of Murcia (IMIB-Arrixaca), Murcia, Spain; ^4^Department of Surgery, Hospital Clínico Universitario Virgen de la Arrixaca, Murcia, Spain; ^5^Department of gastroenterology, Unit of Hepatology, Hospital Clínico Universitario Virgen de la Arrixaca, Murcia, Spain

**Keywords:** citation, regulatory T cells, dendritic cells, chimerism, adoptive transfer, co-occurrence

## Abstract

**Background:**

Operational tolerance in liver transplantation (OT-LT), defined as the graft survival with normal function in absence of immunosuppression, has been a field of intense research since the 1980s. Thereafter, tens of clinical trials and hundreds of articles have been published, making it challenging for researchers to assimilate all the information, more so outside of their disciplines. The aim of the present study was to analyze the research in OT-LT through a new web tool (https://tolerance.imib.es).

**Methods:**

We have developed a web resource that allowed the identification of the present trends and potential research avenues in OL-LT, an overview biomedical terms that were most often cited, including which journals published the most articles, and an advanced search engine that exploited all the information in these publications.

**Results:**

A total of 734 studies were analyzed until November 2023, with a mean of 15 articles published per year, a total sum of 3,751 impact factor points and a total of 26,542 citations. The analysis of citations allowed us to establish a ranking of the most prolific countries, authors, journals and institutions, in addition to the most influential publications in OT-LT. Likewise, keyword and co-occurrence analyses answered which themes involving OT-LT are the most popular, whereas cooperation analysis showed that principal authors in OT-LT form a network, although the lack of international cooperation, especially with regard to clinical trials, appears to be one of the main challenges.

**Conclusion:**

Despite its limitations, our web tool will allow both OT-LT expert and novel researchers to be able to draw a comprehensive picture of the past, present and future of OT-LT research.

## 1 Introduction

Advances in immunosuppressive treatment have had a great impact on the progress and success of solid organ transplantation. The use of immunosuppressive drugs, mainly after azathioprine replacement by cyclosporine in 1979, is effective in preventing and treating graft rejection (Starzl and Fung, 2010). However, the main long-term goals of therapy are to reduce drug exposure while maintaining a well-functioning graft. In this way, the achievement of immune tolerance to an allogeneic donor, defined as the graft survival with normal function in absence of immunosuppression (IS), has been a field of intense research, fueled by a critical need to avoid IS-related side effects (particularly nephrotoxicity, cancer, and cardiovascular events) (Mastoridis et al., 2017). In this regard, liver is defined as an immune-privileged organ (Forrester et al., 2008). While the concept of tolerance to a strange body has been recognized since the early 1950s (Billingham et al., 1953), liver transplantation (LT) tolerance has been addressed in clinical studies since the 1990s (Browne and Kahan, 1994), and although clinical tolerance existence or benefit has been discussed from the beginning (Riordan and Williams, 1999), tens of clinical trials and hundreds of papers, including many reviews, have been published so far. Bibliometric studies are of great help in addressing topics with a huge bibliographic load, providing an overview of the field and helping identify trends and key contributors in the area of interest (Zupic and Cater, 2014). However, no bibliometrics or citation analysis in the field of operational tolerance (OT) in LT (OT-LT) is available worldwide.

The aim of the present study was to analyze the research on OT-LT through a new web resource, to evaluate the progress in this field and try to answer important research questions such as *a)* what is the current publication trend, *b)* which are the most influential articles or authors and *c)* what is the current state of collaboration in OT-LT. Moreover, we highlight the impediments that exist facing current research in this field, and some areas involving OT-LT that may lead the way for the future in this challenging field.

## 2 Materials and methods

### 2.1 Search strategy

We performed a systematic review of scientific publications in the areas of IS withdrawal and OT-LT. All relevant articles were searched without date limits using the PubMed (via MEDLINE) database. Search terms included a combination of standardized index terms: ((tolerance[Title/Abstract]) OR (withdrawal[Title/Abstract]) OR (tapering[Title/Abstract])) AND ((liver transplant^*^[Title/Abstract]) OR (liver graft^*^[Title/Abstract]) OR (liver allograft^*^[Title/Abstract]) OR (hepatic allograft^*^[Title/Abstract]) OR (hepatic transplant^*^[Title/Abstract]) OR (hepatic graft^*^[Title/Abstract])). These keywords were defined by consensus among authors. The search was completed in November 2023.

### 2.2 Selection criteria

The search was limited to studies published in English. Only original articles and reviews were included. Abstracts or book chapter were not considered. Not all items retrieved were suitable for the topic, and for reducing the risk of bias, three researchers independently reviewed them following a selection process with two steps: (a) title screening and (b) abstract screening. The outcomes were filtered to include only those related to OT-LT until 2023. Articles were removed from the program when at least two authors did not accept their suitability. Clinical trials were retrieved in a similar way from ClinicalTrials.gov and the EU Clinical Trial Register databases.

### 2.3 Analysis methods

To determine the structure of research on OT-LT, we developed a new web resource with free access upon reasonable request (https://tolerance.imib.es) that offered the following services: (i) a comprehensive control panel that allowed the identification of the present trends and potential research avenues in OL-LT, including the geographical distribution of publications; (ii) an overview biomedical terms (diseases, chemicals, drugs, genes, or species) that were most often cited, including which journals published the most articles; and (iii) an advanced search engine that exploited all the information in these publications. Notably, in addition to analyzing the scientific aspects of the publications, our approach considered bibliometric indicators, such as the journal impact factor, the journal classification in scientific areas, and the number of citations of the concerned publication.

### 2.4 Web resource development

**Figure 1:**
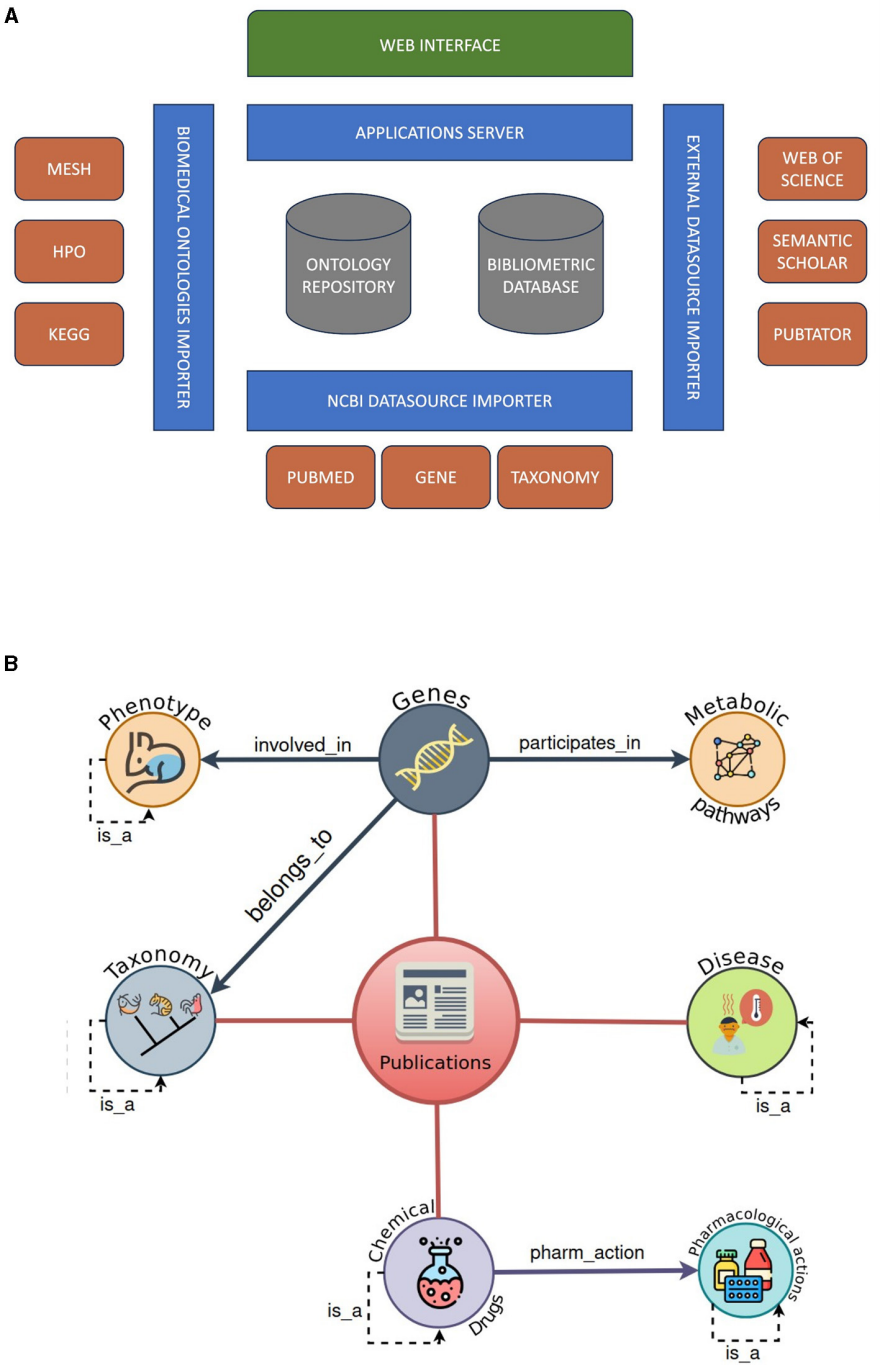
Methodology software platform. **(A)** Web resource architecture. **(B)** Graphical representation of the semantic data model for scientific article classification.

This approach is based on our previous experience in the use of semantic technologies for the use and exploitation of biomedical data (Fernández-Breis et al., 2013; Legaz-García et al., 2015; Esteban-Gil et al., 2017, 2019). Figure 1A shows an outline of the technological architecture developed. This architecture is based on two database management systems: (a) an ontology repository in RDFS based on Virtuoso and (b) the database in which the publications and their metadata (authors, affiliations, tags, etc.) are stored based on MongoDB. On top of these data schemas there are four modules developed with JAVA. Three of these services are in charge of importing data to the databases that we described previously. And the fourth one is in charge of delivering data to the web interface layer (developed in XHTML, Javascript and CSS) to which each user accesses publicly. Each importer module is described as follows: (1) External Datasource Importer is responsible for importing data from source models that do not have a common interface such as Web of Science, Semantic Scholar and Pubtator; (2) NCBI Datasource Importer uses the NCBI public interface to retrieve the publications (PubMed), and once we have annotated them (using Pubtator), we retrieve the information related to the genes and taxonomies discussed in those scientific articles; and (3) Biomedical Ontologies Importer automatically updates the ontologies (MESH, HPO and KEGG) that we use to enrich the information of the publications every 3 months. Once we have defined the software modules that make up the platform, we will define the phases of retrieval and enrichment of the stored bibliometric data. In the first phase, the NCBI import module searches for and downloads OL-LT related articles. The result of this phase is that we have a list of PubMed codes that meet our search criteria, but we do not yet have the publications as such. In a second phase we use these PubMed identifiers to retrieve the publications and all their associated metadata. Once we have downloaded the publications we use Pubtator (Wei et al., 2013) as a text mining tool to note down the following concepts from the articles: diseases, drugs, genes, chemicals, and species. In this process, we separate the title and abstract annotations from the rest of the text in the articles. In this way, we give the user the chance to use full-text annotations or only title-abstract annotations because we cannot annotate the full text of publications that are not open access. From the annotations that Pubtator returns, we use the rest of the resources that we have imported to semantically enrich those annotations. In this phase we have two types of enrichment, we use different approaches: (i) diseases, chemicals, and drugs are enriched using Medical Subject Headings (MeSH) (Coletti and Bleich, 2001); (ii) From the drugs extracted as a concept, we also recovered their pharmacological action; (iii) Genes enriched with the Gene NCBI database (Maglott et al., 2005), Metabolic pathways Kyoto Encyclopedia of Genes and Genomes (KEGG) database (Kanehisa and Goto, 2000) and Human Phenotype Ontology (HPO) (Kohler et al., 2019); and (iv) Species enriched with the Taxonomy NCBI database (Federhen, 2012); and (v) An indirect enrichment is carried out taking advantage of the annotation of the genes, in order to infer metabolic pathways using Kyoto Encyclopedia of Genes and Genomes (KEGG) database (Kanehisa and Goto, 2000) or clinical phenotypes using Human Phenotype Ontology (HPO) (Kohler et al., 2019). Figure 1B represents a graphical diagram of the final classification and enrichment result of each publication. Finally, information from the Semantic Scholar (AI2, 2018) and the Journal Citation Report (JCR) was included in order to classify articles by quality indexes such as the number of citations or impact factor. Furthermore, with this approach, our platform can classify publications in research areas based on journal sources.

## 3 Results

### 3.1 Current publication trends in OT-LT

More than 2,000 references were retrieved from the PubMed database. After the screening phase, 734 studies were included, with a mean of 15 articles published per year, a total sum of 3,751 impact factor points, and a total of 26,542 citations. Forty-one percentage of the articles were published in first-quartile journals (32% in the first decile). The bulk of the OT-LT publications started in 1980, increased progressively to reach their maximum productivity in the last decade (2010s; *n* = 325), projecting a potential surpassing more than 400 publications for the current decade (Supplementary Figure S1), and accounted for 33% of publications in the last 5 years. A total of 194 articles (26%) were reviews. The 32% of these papers were published in the United States (US), followed by China (18%), the United Kingdom (UK) (15%), Japan (14%), Spain and Australia (8%) (Table 1). However, after analyzing the number of publications per million inhabitants, Belgium appeared to be the most productive country, ahead of Australia and UK (Table 1). The first journal in the ranking of the most cited scientific journals is *Transplantation*, with 4,619 citations and 90 publications on OT-LT, followed by *Hepatology* (2,549; 23), the *American Journal of Transplantation* (2,398 citations; 41 publications), *Liver Transplantation* (1,554; 50), *and Transplantation Proceedings* (985; 78) (Table 2). *Journal of Immunology, Nature, Transplant Immunology, Journal of Hepatology* and *Nature Reviews Immunology* complete the list of the top 10 cited journals. The list of most prolific Institutions were headed by the Pennsylvania Commonwealth System of Higher Education (PCSHE) (US), followed by the University of Sydney (Australia), University of California System (US), Centro de Investigación Biomédica en Red (Spain), and London University (UK) (Table 3). When ordered by the number of citations, the University of Rome Tor Vergata (Italy) raised to the seventh position (Table 3). A. Sánchez-Fueyo led the list of the most prolific authors in OT-LT with 44 publications, followed by A.W. Thomson, G. A. Bishop, S. Goto, and A.J. Demetris (Table 4). While Sánchez-Fueyo published his first OT-LT paper in 2002 (Sánchez-Fueyo et al., 2002), the other four authors began their contributions approximately 10 years prior (Supplementary Figure S2). The classification changed when analyzed by citation number, therefore T. E. Starzl (*n* = 2,199), Martinez-Llordella (*n* = 1,674), Qian (*n* = 1,564), and G. Tissone (*n* = 1,486) moved into the top 10 list of most cited authors (Table 4).

**Table 1:** Top countries in OT-LT research.

**Rank**	**Country**	**Publications**	**Publications/million inhabitants**	**LT/year/million inhabitants^a^**
1	US	234	0.70 [6]	28.5 [1]
2	China	133	0.08 [10]	4.2 [9]
3	UK	110	1.62 [3]	13.5 [6]
4	Japan	107	0.83 [5]	3.4 [10]
5	Spain	57	1.23 [4]	24.8 [4]
6	Australia	57	2.26 [2]	9.9 [7]
7	Germany	53	0.62 [7]	8.9 [8]
8	Belgium	39	5.05 [1]	25.4 [2]
9	France	33	0.47 [8]	19.7 [5]
10	Italy	24	0.40 [9]	24.5 [3]

The number in brackets indicates the position that countries would rank.

^a^Data for 2022 obtained from Global Observatory on Donation and Transplantation (http://www.transplant-observatory.org).

LT, liver transplantation.

**Table 2:** Top journals in OT-LT research.

**Rank**	**Journal**	Citations^*^	**Publications**
1	Transplantation	4,619	90 [1]
2	Hepatology	2,549	23 [7]
3	American Journal of Transplantation	2,398	41 [4]
4	Liver Transplantation	1,554	50 [3]
5	Transplantation Proceedings	985	78 [2]
6	Journal of Immunology	877	8
7	Nature	803	1
8	Transplant Immunology	801	36 [5]
9	Journal of Hepatology	748	11
10	Nature Reviews Immunology	654	1
11	The Lancet	585	3
12	Frontiers in Immunology	579	29 [6]
13	The Journal of Clinical Investigation	506	3
14	Nature Medicine	465	3
15	Pediatric Transplantation	461	18 [8]
16	Human Immunology	403	12
17	Seminars in Liver Diseases	379	5
18	Immunological Reviews	370	3
19	Immunology	363	6
20	Transplant International	339	14 [10]
21	Current Opinion in Organ Transplantation	335	18 [9]

^*^Semantic Scholar Citations. The number in brackets indicates the position that journals would rank.

**Table 3:** Top organizations in OT-LT research.

**Rank**	**Research Organization**	**Publications**	Citations^*^
1	Pennsylvania Commonwealth System of Higher Education (US)	77	5,579 [1]
2	University of Sydney (AUSTRALIA)	36	1,825 [3]
3	University of California System (US)	34	1,514 [6]
4	Centro de Investigacion Biomedica en Red-CIBERehd (SPAIN)	32	2,149 [2]
5	University of London (UK)	29	1,529 [5]
6	Universite Catholique Louvain (BELGIUM)	27	1,691 [4]
7	King's College Hospital NHS Foundation Trust (UK)	21	1,446 [8]
8	Harvard University (US)	25	1,403 [9]
9	Kyoto University (JAPAN)	24	1,401 [10]
10	Assistance Publique Hopitaux Paris (FRANCE)	20	660
11	Chang Gung Memorial Hospital (CHINA)	18	261
12	Northwestern University (US)	18	839
13	University of Oxford (UK)	18	1,198
14	University of Pennsylvania (US)	16	734
15	University of Barcelona (SPAIN)	16	947
16	National Center for Child Health & Development (JAPAN)	14	346
17	Stanford University (US)	14	501
18	Zhejiang University (CHINA)	14	125
19	Chongqing Medical University (CHINA)	13	258
20	University of Rome Tor Vergata (ITALY)	12	1,471 [7]

^*^Semantic Scholar Citations. The number in brackets indicates the position that institutions would rank.

**Table 4:** Top authors in OT-LT research.

**Rank**	**Author**	**Publications**	Citations^*^
1	Sanchez-Fueyo, Alberto	44	3,019 [1]
2	Thomson, Angus W	40	2,773 [3]
3	Bishop, G Alex	27	1,540 [8]
4	Goto, Shigeru	26	453
5	Demetris, Anthony J	25	2,847 [2]
6	McCaughan, Geoffrey W	23	1,418 [10]
7	Feng, Sandy	21	934
8	Calne, R Y	20	1,221
9	Fung, John J	19	1,865 [5]
10	Kamada, N	18	615
11	Uemoto, Shinji	17	963
12	Chen, Chao-Long	17	257
13	Qian, Shiguang	16	1,564 [7]
14	Mazariegos, George V	15	1,185
15	Starzl, Thomas E	15	2,199 [4]
16	Martinez-Llordella, Marc	15	1,674 [6]
17	Li, Wei	14	966
18	Lord, Roger	13	233
19	Levitsky, Josh	12	415
20	Londono, Maria-Carlota	12	910
21	Lu, L	11	1,219
22	Pons Miñano, Jose Antonio	11	726
23	Koshiba, Takaaki	11	929
24	Tisone, Giuseppe	10	1,486 [9]
25	Baroja-Mazo, Alberto	10	318

^*^Semantic Scholar Cites. The number in brackets indicates the position that authors would rank.

### 3.2 The most influential articles on OT-LT

The most cited article in OT-LT was the first publication in the area as far back as 1969, carried out in porcine model (Calne et al., 1969), followed by a review about hepatic T cells and tolerance published in 2003 (Crispe, 2003). The median year of publication for the five most cited publications was 1991 (range, 1969–2003) (Table 5). However, when we analyzed the citation velocity or the influential citation count score (AI2, 2018), the median year of publication raised to 2017 and 2011, respectively (Table 5). None of the five most cited publications appeared among the five publications with the highest citation velocity, and only one (Crispe, 2003) appeared in the influential citation count score list (Table 5). Likewise, two publications based in a pilot study using adoptive transfer of Tregs (Todo et al., 2016) and the modulation of liver tolerance mediated by antigen-presenting and regulatory cells (Horst et al., 2016) appeared in both the citation velocity and influential score lists (Table 5).

**Table 5:** Top publications in OT-LT research using semantic scholar citation scores.

**Rank**	**Publication**	**PMID**	**Author**	**Journal**	**Year**	**Citations**	**Citation Velocity**	**Influential Citation Count**
1	Induction of immunological tolerance by porcine liver allografts	4894426	Calne et al.	Nature	1969	800	22	5
2	Hepatic T cells and liver tolerance	12511875	Crispe et al.	Nat. Rev. Immunology	2003	640	21	21
3	Murine liver allograft transplantation: tolerance and donor cell chimerism	8138266	Qian et al.	Hepatology	1994	466	8	2
4	Weaning of immunosuppression in liver transplant recipients	9020325	Mazariegos et al.	Tranplantation	1997	412	9	5
5	Systemic chimerism in human female recipients of male livers	1357298	Starzl et al.	The Lancet	1992	373	4	8
**Rank**	**Publication**	**PMID**	**Author**	**Journal**	**Year**	**Citations**	**Citation Velocity**	**Influential Citation Count**
1	A pilot study of operational tolerance with a regulatory T-cell-based cell therapy in living donor liver transplantation	26773713	Todo et al.	Hepatology	2016	289	42	10
2	Applicability, safety, and biological activity of regulatory T cell therapy in liver transplantation	31715056	Sanchez-Fueyo et al.	Am. J. Transplantation	2019	96	31	7
3	Liver mediated adaptive immune tolerance	31787967	Zheng et al.	Frontiers Immunol.	2019	111	30	2
4	Modulation of liver tolerance by conventional and nonconventional antigen-presenting cells and regulatory immune cells	27041638	Horst et al.	Cell. Mol. Immunol.	2016	174	28	9
5	Immune tolerance in liver disease	24913836	Crispe IN.	Hepatologty	2014	76	25	12
**Rank**	**Publication**	**PMID**	**Author**	**Journal**	**Year**	**Citations**	**Citation Velocity**	**Influential Citation Count**
1	Hepatic T cells and liver tolerance	12511875	Crispe et al.	Nat. Rev. Immunology	2003	654	17	23
2	Immune tolerance in liver disease	24913836	Crispe IN.	Hepatologty	2014	76	25	12
3	Using transcriptional profiling to develop a diagnostic test of operational tolerance in liver transplant recipients	18654667	Martínez-Llordella et al.	JCI	2008	308	8	11
4	A pilot study of operational tolerance with a regulatory T-cell-based cell therapy in living donor liver transplantation	26773713	Todo et al.	Hepatology	2016	316	42	10
5	Modulation of liver tolerance by conventional and non-conventional antigen-presenting cells and regulatory immune cells	27041638	Horst et al.	Cell. Mol. Immunol.	2016	199	28	9

PMID, PubMed identifier.

### 3.3 Keywords and co-occurrence analysis

**Figure 2:**
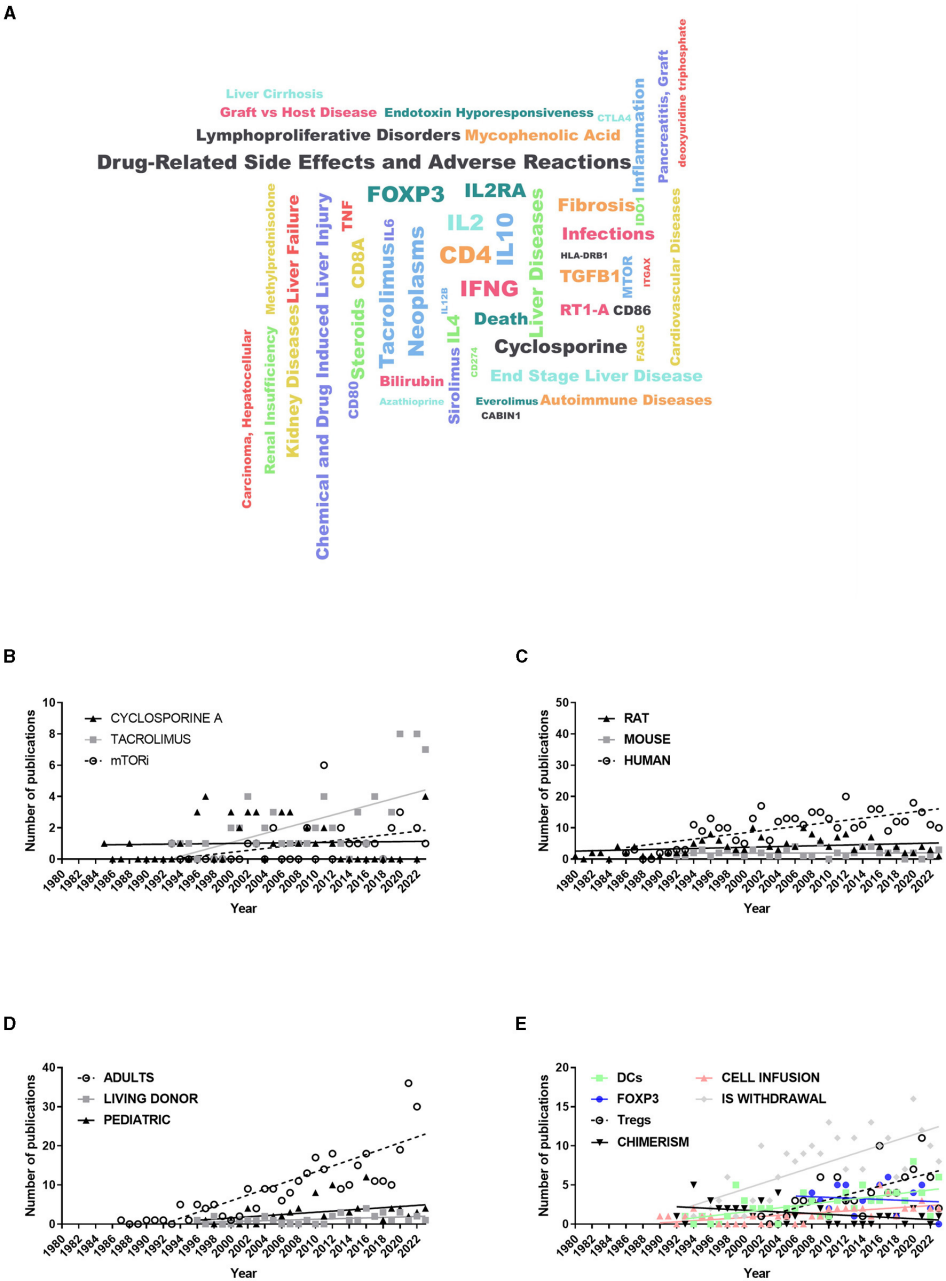
Keyword analysis in OT-LT research. **(A)** Word Cloud representation of the most popular genes, drugs/chemicals or liver transplant-related diseases among the 664 OT-LT publications selected in the study. **(B–E)** Timeline of the most relevant terms in OT-LT research, grouped by IS drug **(B)**, including cyclosporine A, Tacrolimus and inhibitors of mTOR **(B)**; study model, including rat, mouse and human **(C)**; by type of transplantation, including adult, pediatric or living donor liver allograft **(D)**; or by different keywords, including dendritic cells (DCs), regulatory T cells (Tregs), chimerism, Forkhead Box P3 (FOXP3), cell adoptive transfer or immunosuppression (IS) withdrawal **(E)**. All keywords were retrieved exclusively from the title or abstract of the selected publications. A linear regression was drawn for all terms to know the trend along time.

We extracted the keywords from the 734 papers and grouped them into three clusters based on LT-related diseases, drugs/chemicals, and genes. When we analyzed the most popular words in OT-LT (Figure 2), we noted that CD4, Interferon-γ (IFNG), Forkhead box P3 (FOXP3), Interleukin (IL)-2RA and IL-10 stood out among genes and even among all keywords put together (Figure 2A; Supplementary Table S1). Regarding diseases, neoplasms and drug-related side effects appeared to be the most important problems associated with OT-LT research. Furthermore, tacrolimus and cyclosporine A (CsA), both calcineurin inhibitors (CNIs), were the most representative immunosuppressant drugs, ahead of steroids or mammalian Target of Rapamycin (mTOR) inhibitors (sirolimus or everolimus). Moreover, CsA has maintained a stable level of publications over the years, despite the appearance of new drugs, which have progressively increased (Figure 2B). Notably, all these keywords had huge co-occurrences (Supplementary Figure S3), indicating the important relationship among drugs, LT-related diseases, and genes or proteins studied in the OT-LT publications.

When we analyzed keywords by research model, we observed that 178 articles (excluding reviews) used rats as LT model, and other 64 used mice. The OT-LT rat-model has been carried out continuously over time, similar to the murine-model research (Figure 2C). However, the number of human studies has increased continuously since the first reported event in 1987 (Mohanakumar et al., 1987). Likewise, 10% of publications studied OT-LT in the pediatric population and 5% were conducted on living donor LT. Moreover, while adult OT-LT research has been growing in strength since the first publications in the late 1980s, the number of OT-LT publications in children or with living donors has remained constant or increased slightly since the mid-1990s (Figure 2D). Moreover, other keywords shine with their own light, including 58 publications regarding “chimerism”, 200 publications performing IS “withdrawal”, 90 which appoint the “regulatory T cells” (Tregs) function, while 51 indicate the nuclear factor Forkhead box P3 (“FOXP3”) as a biomarker. In addition, 63 articles centered on the tolerogenic action of “dendritic cells” (DCs), and 45 articles studied the “adoptive transfer” of different kinds of cells (mesenchimal stem cells (MSCs), DCs, or Tregs). After analyzing the temporal evolution of these keywords (Figure 2E), we found that almost all of them grew over time, specifically IS withdrawal and Tregs. However, the use of chimerism dropped significantly, although it was one of the words that first appeared in the timeline. FOXP3 appeared as an exceptionally constant term during the last 15 years, with an average of more than three publications per year. On the other hand, an inter-specific analysis of different keywords by research model shows that CD4, IL-2, FOXP3, IFNG, and IL-10 were the most coincident genes (Supplementary Figure S4A), together with tacrolimus, cyclosporine, and steroids in the cluster of drugs. After separating studies on children from those on adults, we found that most of the genes were shared with those revealed in the inter-specific analysis, and only CD8a appeared among the five most coincident genes instead of IFNG (Supplementary Figure S4B). In this case, the three most coincident LT-related diseases were drug-related side effects, fibrosis, and neoplasms (Supplementary Figure S4B).

### 3.4 Cooperation analysis

**Figure 3:**
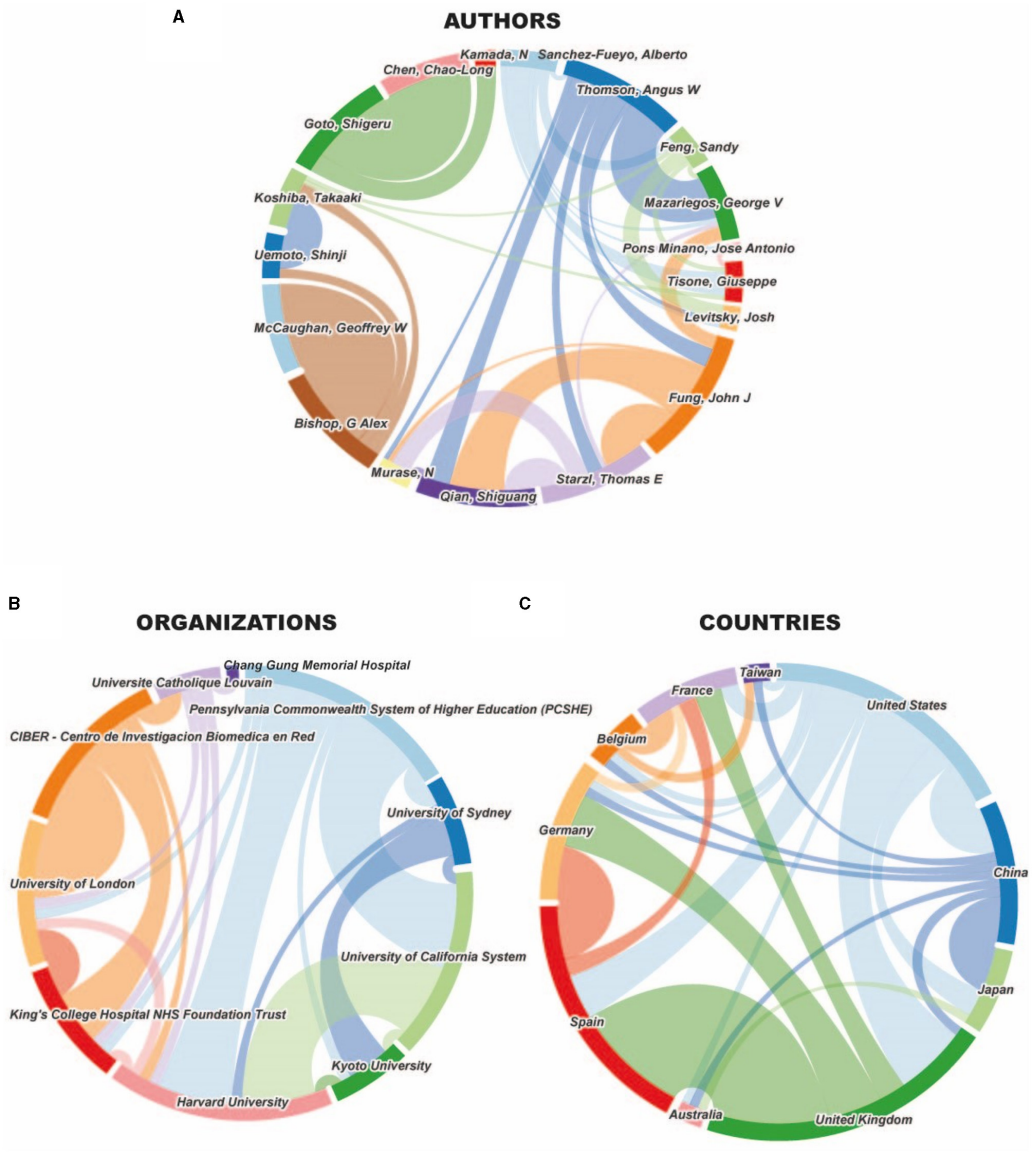
Cooperation in OT-LT research. Wheel chart showing the interactions among the 18 most prolific principal authors **(A)**, the 10 most important institutions **(B)** and the 10 most significant countries **(C)** in OT-LT research.

To establish a ranking of collaborations, we studied the level of cooperation among authors, institutions, and countries (Figure 3). We found that after selecting the most prolific authors that we considered principal investigators in OT-LT, the level of collaboration was apparently high among them (Figure 3A). Detailed analysis showed that the cooperation level was not completely cross-sectional. We could divide most of the authors into four main nodes; the “Pittsburgh/Dr. Starzl” node including Thomson, Fung, Mazariegos, Qian and Murase; the “Sidney” node including McCaughan and Bishop; “Dr. Kamada” node including Goto and Chen; and the “Kyoto” node including Uemoto and Koshiba. Taking this into account, cooperation was practically intra-nodal, with a few exceptions. The remaining authors had more inter-nodal collaborations, mainly Dr. Sanchez-Fueyo, who published a third of his articles with six authors from external institutions, including Drs. Feng, Pons, Tisone, Levitsky, and the Pittsburgh node. However, after analyzing the relationship among the ten institutions (Figure 3B) or countries (Figure 3C) with a higher number of publications in OT-LT research, we observed a broad collaboration, with a mean of 4.6 (range, 1–8) and 4.7 (range, 3–8) relations per organization and country, respectively. The number of publications per node was highly variable, with highest numbers of 8 and 13 collaborations between the University of California and PCSHE and between Spain and Australia, respectively. These collaborations represented 44% of articles published for the selected centers but only 25% for countries.

### 3.5 Clinical trials

**Figure 4:**
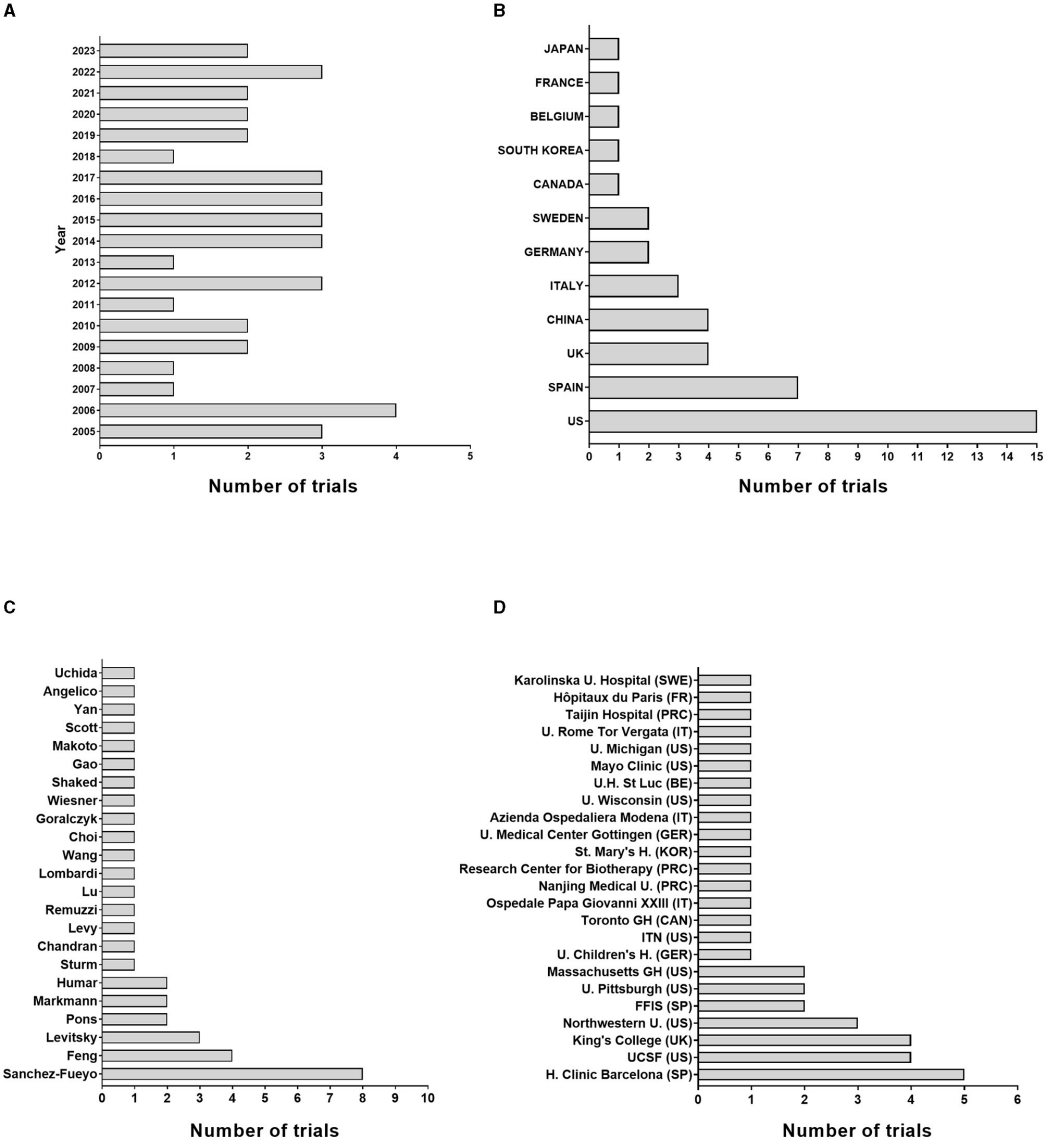
Clinical trials in OT-LT research. Bar graph showing the number of trials per year **(A)**, country **(B)**, author **(C)** institution **(D)**. UCSF, University of California San Francisco; FFIS, Fundación para la Formación e Investigación Sanitaria de la Región de Murcia; ITN, Immune Tolerance Network.

Since 2005, 42 clinical trials have been registered in the US and European Union official registers addressing OT-LT (Supplementary Table S2), with a mean of almost two trials per year (Figure 4A). Clinical trials have been performed in 12 countries, with the US having the highest number of trials (*n* = 15), followed by Spain (*n* = 7), the UK and China (*n* = 4), and Italy (*n* = 3); moreover, Sweden and Germany had two trials each (Figure 4B). Six researchers have led more than 1 trial on OT-LT, and Dr. Sánchez-Fueyo heads the list (*n* = 8), followed by Dr. Feng (*n* = 4), Dr. Levitsky (*n* = 3) and Drs. Pons, Markmann, and Humar with two trials each (Figure 4C). Likewise, seven institutions hosted several trials, including the Hospital Clinic de Barcelona (Spain, *n* = 5), University of California San Francisco (US, *n* = 4), King's College (UK, *n* = 4), Northwestern University (US, *n* = 3) and Fundación para la Formación e Investigación Sanitaria de la Región de Murcia (Spain), University of Pittsburgh (US) and Massachusetts General Hospital (US) with two trials each (Figure 4D). Only ten trials were multicentric, and none of these included institutions from different countries (Supplementary Table S2).

## 4 Discussion

In the clinical setting, OT is defined as the absence of acute and chronic rejection, and graft survival with normal function and histology in an IS-free, fully immunocompetent host, usually as an end result of a successful attempt at IS withdrawal (Wieers et al., 2007). The first OT-LT study dates back to 1969 (Calne et al., 1969), but it was not until the 1980s that the bulk of OT-LT research began, mainly in rodent models (Houssin et al., 1980; Kamada et al., 1980). Thenceforth, OT-LT has been a field of intense research, fueled by a critical need to avoid IS-related side effects. The huge amount of life science literature and associated knowledge available online makes it challenging for researchers to assimilate all the useful information, more so outside of their disciplines. This has generated both the demand and opportunity for the development of advanced computer-based word-processing tools to support traditional information-processing systems (Hristovski et al., 2013). We have developed a new web resource to facilitate the search of scientific literature and the exploitation of all information associated with these publications. Therefore, we present a comprehensive analysis of OT-LT to evaluate the progress in the field and answer important research questions.

The continuous increase in the number of publications in the last two decades is perfectly suited to the sharp increase in the number of studies conducted on humans since the early 1990s, principally adults, and with the implementation of controlled protocols of IS withdrawal. OT-LT has attracted the attention of researchers, as indicated by contributions from the most important countries. Obviously, the top publishing institutions belong to nations where transplant programs are widely implemented (Trotter, 2017). However, the ranking did not fit perfectly; some countries with a high rate of LT per million inhabitants, had a small number of OT-LT research articles (e.g., Italy and France). China and Japan showed opposite findings, probably due to their overwhelming populations or even by the high levels of investment in research compared to other countries (OECD, 2023). OT-LT belongs to a broad area of research, and as expected, the most influential journals in OT-LT were specialized in transplantation, hepatology, and also immunology. It is noteworthy how prestigious journals such as *Nature* or *The Lancet*, rank the top positions in number of citations with only two or three publications, respectively. Furthermore, most of these journals have a high JCR index; therefore, OT-LT has received attention from some of the best journals in the field of transplantation, whereas the leading publications have citation rates similar to those of more prolific research areas.

Although several methods are available to measure the impact of a research publication, citation analysis is the most common (Ding and Cronin, 2011). However, this method may be biased, as older publications may be favored. Thus, the use of new metrics is necessary to understand the current trend and the most influential publications in OT-LT research. “Citation Velocity” is a weighted average of the publication's citations for the last 3 years and fewer for publications published in the last year or two, which indicates how popular and lasting the publication is (AI2, 2018). Likewise, “Influential Citation Score” is determined utilizing a machine-learning model analyzing a number of factors including the number of citations to a publication, and the surrounding context for each, identifying citations where the cited publication has a significant impact on the citing publication, making it easier to understand how publications build upon and relate to each other (AI2, 2018). In this context, we can observe that animal models have lost influence in OT-LT research. Although the rat-model LT is a well-established experimental model with hundreds of publications in the last 15 years, rodent models in OT-LT research are usually based on the spontaneous ability to tolerate a liver from a different haplotype (Yokota et al., 2016). This makes it difficult to use this model to emulate IS withdrawal protocols carried out with patients. On the other hand, the idea of chimerism, which began in the early 1990s, has lost its strength; moreover, some publications have pointed out that chimerism does not influence allograft tolerance in human LT after IS withdrawal (Pons et al., 2003). However, we can highlight as a current trend studies that attempt to explain the immunological causes leading to OT-LT (Crispe, 2003), IS withdrawal protocols (Benitez et al., 2013), as well as adoptive cell transfer trials (Todo et al., 2016). Strikingly, a publication on living donors and pediatric transplantation (Feng et al., 2012) appeared as one of the most influential publications, even though this topic represented a limited percentage of the publications in this area. However, it is clear that this is a subject of great interest to LT researchers.

Tregs and DCs have been implicated in OT-LT in the last decades (Baroja-Mazo et al., 2016a). Tregs promote a state of antigen-specific peripheral tolerance by suppressing the activation and expansion of reactive effector cells. In 1970, it was found that T-cells not only amplified but also dampened immune responses and that this downregulation was mediated by T-cells that were different from Th-cells (Gershon and Kondo, 1970). The term regulatory or suppressor cells was reintroduced in 1995 based on studies that reported the development of a lethal autoimmune disease in mice thymectomized in the neonatal period (Sakaguchi et al., 1995). Tregs are characterized by the CD25 (IL2RA) and the FOXP3 expression, two of the most commonly used terms in OT-LT. *FOXP3* was initially identified as the gene responsible for an X-linked recessive inflammatory disease in scurfy mutant mice, as well as fatal autoimmune/inflammatory disease, immune dysregulation, polyendocrinopathy, enteropathy, or X-linked syndrome in humans (Brunkow et al., 2001). The essential role of FOXP3 underlines the crucial importance of naturally arising FOXP3^+^CD4^+^Tregs for self-tolerance and immune homeostasis (Haque et al., 2011). Although the specific mechanism of action of Tregs is still not completely known, several studies have shown, for example, that Tregs use different mechanisms to hamper effector T-cells (Teffs): the modulation of dendritic cell function, IL-2 deficit, direct cytotoxicity, or the secretion of inhibitory cytokines, such as IL-10, IL-35, or tumor growth factor (TGF)-β (Pons et al., 2013). DCs are also involved in establishing immune tolerance by deleting T-cell clones or inducing Tregs (Zhuang et al., 2020). Typical tolerogenic DCs secrete and express cell surface regulatory molecules that mediate tolerogenic effects on recipient T-cells. As a result, tolerance to the donor antigen can be achieved through induction of Tregs as well as via the induction of anergy and deletion of effector and memory T-cells (Ezzelarab and Thomson, 2011). Nevertheless, the fact that both cells continue to be among the most frequently mentioned terms seem to be due to the importance of the adoptive transfer trials that have been growing in recent years. For example, clinical trials have been conducted infusing *in vitro* expanded Tregs (Yu et al., 2020), DCs (Que et al., 2020), or MSCs (Vandermeulen et al., 2022) and replacing classic immunosuppressive drugs with rapamycin derivatives (Adams et al., 2015), or even based on the differential effect exerted by Inhibitors of mechanistic Target of Rapamycin (mTOR) on human Tregs and Teffs. These drugs are used in solid organ transplantation to induce IS while promote the expansion of Tregs, which could lead to immunological tolerance (Baroja-Mazo et al., 2016b). CNIs, particularly tacrolimus, are first-line immunosuppressive drugs in most LT centers, both in Europe and the US (Di Maira et al., 2020). One of the major adverse effects of CNIs is nephrotoxicity, which leads to renal replacement therapy and increases mortality risk (Ojo et al., 2003). Therefore, other immunosuppressive regimens have been implemented, such as sirolimus or everolimus, which are potent immunosuppressive drugs with comparable efficacies, and could also better optimize renal function and diminish side effects compared with CNIs (Baroja-Mazo et al., 2016b).

It is also interesting to note how the co-occurrence of words highlights a great similarity between results obtained in rat and human models. Rat LT is a well-established experimental model (Yagi et al., 2013) with hundreds of publications in the last 15 years. Moreover, preclinical studies with rodents have been accepted as a previous step in clinical trials (Bryda, 2013). Nevertheless, both species share 95% of their genes (Gibbs et al., 2004). Keyword co-occurrence occurs when two keywords appear together in an article, indicating that a relationship exists between both concepts. Keyword analysis is conducted because an author's keywords adequately characterize an article's content. Scientific researchers use co-occurrence analyses to measure performance and draw innovations and information flows (Wormell, 2000).

Collaboration among researchers is the most recognized method of intellectual association in scientific research (Cisneros et al., 2018). The convergence of individual perspectives leads to the evolution and development of ideas. Moreover, a paper published by multiple authors has a more improved quality because fewer mistakes are made and contributions are provided from diverse disciplines (Tahamtan et al., 2016). Principal authors in OT-LT form a network, which suggests that research concentrates on a few authors, and most of the nodes appear to form a network of two or three. The co-authorship network can, thus, be seen as a collection of few networks that is fairly closed and shows few interactions among themselves. However, some authors such as Sánchez-Fueyo, Feng, and Thomson play an important role in the network, acting as knowledge brokers among groups. Notably, several researchers have followed the path paved by the leader and have established their own lines of research in OT-LT. This can also be applied to clinical trials, where, unfortunately, there are no multi-center trials conducted in different countries, even in the same continent. Understanding this issue is challenging. In recent years, Europe has hosted almost 3,000 multinational trials annually (https://www.clinicaltrialsregister.eu/). The limited number of groups studying clinical LT-OT, the complexity of proposed trials involving strict IS weaning protocols or high-tech expanded cell infusion, as well as variations in legislation, could all influence the results we observe.

Nevertheless, there are some limitations that may be biasing this study. We use PubMed and Semantic Scholar as the main sources for article retrieval and citation, respectively. The use of other bibliographic and citation sources, could improve the results. Likewise, older articles do not have the abstract available, so keyword retrieval from those abstracts is not possible. Moreover, non-selection of articles in languages other than English, or studying OT in organs other than liver, may result in the loss of some relevant contribution. We did not discard self-citations, which may be related to a visibility strategy by journals or authors (Gonzalez-Sala et al., 2019), although the analysis of citation based in indicators for 15 fields in the sciences, social sciences and humanities confirmed that, at this level of aggregation, there was no need to revise the national indicators and the underlying journal citation measures in the context of excluding self-citations (Glänzel and Thijs, 2004). Furthermore, we are unable to distinguish whether publications categorized by country originate from Universities, research institutes, or simply Foundations or Associations headquartered there. This lack of distinction may lead to inflated numbers of certain countries.

## 5 Conclusions and future directions

While acknowledging its limitations, our study presents a coherent depiction of OT-LT research through a comprehensive analysis and structured literature review conducted using our proprietary program. This provides both seasoned experts and emerging researchers with insights into the evolution of the field and a visual roadmap for its future direction. Likewise, we are able to detect impediments facing current research in this field despite continuing progress. Collaboration among researchers is necessary to develop a field, and therefore more cross-country collaborations are needed. Moreover, global collaboration networks allow developing nations to engage in the knowledge creation process traditionally led by developed countries (Palacios-Callender and Roberts, 2018). Clinical trials are essential for the development of new treatments, and when well-designed, can benefit the participants, investigators, and the medical community (Novitzke, 2008). This includes the need for a higher number of multi-center clinical trials and collaboration among institutions from different countries and even from different continents. Although women represent up to 60% of students entering the medical profession in many countries in the world (Kilminster et al., 2007), only two women, Dr. Sandy Feng and Dr. Noriko Murase, featured among the most important authors in OT-LT. The participation of both men and women is essential in ventures to create a more humane environment for the training and practice of medicine (Flaherty et al., 2019). Additionally, numerous studies have investigated the genetic and phenotypic profiles of tolerant transplant patients. Moreover, different groups have defined several biomarkers to distinguish between potentially tolerant and non-tolerant patients. However, most of these biomarkers have not practically been reanalyzed in an independent and/or prospective way by other groups (Pérez-Sanz et al., 2019) or even established for clinical practice (Kurian et al., 2018). Thus, more inter-center validation studies will be necessary to overcome this gap between bench and patients.

Furthermore, from our perspective, future research directions in OT-LT may be more focused on an induction of tolerance. This could entail not only exploring regulatory cell infusion trials, incorporating novel CAR-Tregs (Kaljanac and Abken, 2023; Proics et al., 2023), but also using new therapeutic or pharmacological approaches (Baroja-Mazo et al., 2018) or even manipulating aspects such as diet (Wu et al., 2020).

In conclusion, OT-LT remains an exciting frontier within the captivating realm of transplantation, poised to sustain the interest of researchers across diverse disciplines, including surgery, hepatology, immunology, and molecular biology.

## Data availability statement

The original contributions presented in the study are included in the article/Supplementary material, further inquiries can be directed to the corresponding authors.

## Author contributions

ÁE-G: Conceptualization, Methodology, Software, Validation, Writing—original draft, Writing—review & editing. JM-G: Investigation, Writing—review & editing. DV-C: Formal analysis, Writing—review & editing. AM-M: Investigation, Writing—review & editing. PR: Conceptualization, Formal analysis, Supervision, Writing—review & editing. JP: Conceptualization, Formal analysis, Funding acquisition, Methodology, Supervision, Writing—review & editing. AB-M: Conceptualization, Formal analysis, Funding acquisition, Investigation, Methodology, Supervision, Writing—original draft, Writing—review & editing.
