# Supplementary material for: Operational tolerance research in liver transplantation: a bibliometric analysis using a new web resource
Source: Front Res Metr Anal. 2024 Mar 14;9:1368534. doi: 10.3389/frma.2024.1368534 (PMC10973155; doi:10.3389/frma.2024.1368534)
Supplement: Supplementary file 1 [file Data_Sheet_1.pdf]

Operational tolerance research in liver transplantation: A bibliometric analysis using a new open web resource

Ángel Esteban-Gil, Juan José Martínez-García, Daniel Vidal-Correoso, Ana M. Muñoz-Morales, Pablo Ramírez, José Antonio Pons and Alberto Baroja-Mazo.

SUPPORTING INFORMATION DESCRIPTION

Supplementary Tables

Table S1.- Top most named keywords as grouped by related liver transplant diseases, drugs or genes.

Table S2.- Clinical trials in OT-LT registered in ClinicalTrials.gov and the EU Clinical Trial Register.

Supplementary Figures

Figure S1.- Number of publications in OT-LT research over time. Graph bar of the number of articles published about OT-LT as separated by decades**.** The publications anticipated for the 2020s decade have been extrapolated from the data collected between 2020 and 2023.

Figure S2.- Number of publications of the five most prolific authors in OT-LT research along the time.

Figure S3.- Keyword co-occurrence in OT-LT research. Sankey diagram of the co-occurrence of the most cited keywords among the three selected clusters (genes, drugs/chemicals and liver transplant related diseases).

Figure S4.- Keyword analysis by title and abstract. Sankey diagram of the co-occurrence of the most cited keywords among research models (A) and after separating studies on children from those on adults (B).

Table S1.- Top most named keywords as grouped by related liver transplant diseases, drugs or genes

Rank	LT related diseases	Publications	Drugs	Publications	Genes (Symbol)	Publications
**1**	Neoplasms	53	Tacrolimus	43	CD4	63
**2**	DRSE[1]	34	Cyclosporine	32	IL10	59
**3**	Liver Diseases	33	Steroids	27	IFNG	52
**4**	Fibrosis	28	Mycophenolic Acid	16	IL2	51
**5**	CDILI[2]	24	Sirolimus	15	FOXP3	46
**6**	Infections	24	Bilirubin	13	IL4	26
**7**	Kidney Diseases	23	Methylprednisolone	9	IL2RA	26
**8**	Death	22	Everolimus	7	CD8A	25
**9**	Liver Failure	20	D3P[3]	7	TGFB1	22
**10**	Inflammation	20	Sodium Chloride	6	RT1-A	15
**11**	End Stage Liver Disease	19	Azathioprine	6	CTLA4	14
**12**	LPD[4]	18	Iron	5	TNF	14
**13**	Autoimmune Diseases	13	Prednisone	5	MTOR	12
**14**	Renal Insufficiency	13	Lipids	4	CD86	12
**15**	Pancreatitis, Graft	12	Creatinine	4	CD80	12
**16**	Cardiovascular Diseases	11	Lipopolysaccharides	4	IL6	12
**17**	Graft vs Host Disease	11	Basiliximab	4	CD274	9

**18**	Drug Hypersensitivity	10	Glucose	3	IDO1	9
**19**	ET[5] Hyporesponsiveness	10	Mitomycin	3	FASLG	8
**20**	Carcinoma, Hepatocellular	10	Digoxigenin	3	ITGAX	7
**21**	Liver Failure, Acute	9	Cyclophosphamide	3	CABIN1	6
**22**	Hypertension	9	Gadolinium chloride	3	IL17A	6
**23**	Liver Cirrhosis	9	Thymidine	2	CD28	6
**24**	Necrosis	9	Acetaminophen	2	PDCD1	5
**25**	Hepatitis C	8	Cholesterol	2	CD40	5

[1] DRSE, Drug-Related Side Effects and Adverse Reactions

[2] CDILI, Chemical and Drug Induced Liver Injury

[3] D3P, Deoxyuridine triphosphate

[4] LPD, Lymphoproliferative Disorders

[5] ET, Endotoxin

Table S2.- Clinical trials in OT-LT registered in ClinicalTrials.gov and the EU Clinical Trial Register

Title	Database Identifier	Start Date (year)	Institution	Principal Investigator	Multicenter
Efficacy and Safety of Immunosuppressive Withdrawal After Pediatric Liver Transplantation	NCT06147375	2023	Tianjin First Central Hospital (PRC)	Wei Gao	No
Treg Cell Therapy in Liver and Kidney Transplantation - Preclinical Validation of Batches of Treg Cells Amplified in Vitro	NCT04661254	2023	Hôpitaux de Parix (FRANCE)	Miyara Makoto	No
Erythropoietin Therapy to Induce Regulatory T Cells in Liver Transplant Recipients	NCT05325073	2022	Northwestern University (US)	Josh Levitsky	No
A Study of TCD601 in the Induction of Tolerance in de Novo Liver Transplantation	NCT06019507	2022	Karolinska University Hospital (SWEDEN)	Jesse Scott	No
Immune Tolerance After Pediatric Liver Transplantation-a Cohort Study	NCT05501301	2022	RenJi Hosptial (PRC)	Liu Yan	No
A 60 month, single-arm, proof-of-concept study to induce allogeneic tolerance in deceased donor liver transplant recipients using siplizumab, an anti-CD2 antibody in combination with cyclophosphamide and splenectomy	2021-001680-24	2021	IBT-MED AB (SWEDEN)	-	No
A Phase I/II Drug Withdrawal Study of Alloantigen-Specific Tregs in Liver Transplantation	NCT03654040	2021	University of California San Francisco (US)	Sandy Feng	No
De Novo Metabolic Syndrome in Liver Transplant Patients After Immunosuppression Withdrawal	NCT05808192	2020	University of Rome Tor Vergata (ITALY)	Roberta Angelico	No
Phase I/II Study to Evaluate the Safety and Efficacy of JB-101(Induced T Cell With Suppressing Functions), to Induce Operational Tolerance in Living Donor Liver Transplantation	NCT04950842	2020	Juntendo University (JAPAN)	Koichiro Uchida	Yes[a]
A Phase I/II Drug Withdrawal Study of Alloantigen-Specific Tregs in Liver Transplantation (ITN073ST)	NCT03577431	2019	Massachusetts General Hospital (US)	James F. Markmann	No

Safety and Preliminary Efficacy of Delayed Donor-derived Regulatory Dendritic Cell (DCreg) Infusion and Immunosuppression Withdrawal in Living Donor Liver Transplant (LDLT) Recipients	NCT04208919	2019	University of Pittsburgh (US)	Abhinav Humar	No
Multicenter, randomized, prospective study to establish the clinical efficacy and the mechanisms of tolerance following immunosuppression withdrawal in liver transplantation	2017-004983-37	2018	Fundación para la Formación e Investigación Sanitaria de la Región de Murcia (SPAIN)	José Antonio Pons	Yes[b]
Safety and Preliminary Efficacy of Donor-derived Regulatory Dendritic Cell (DCreg) Infusion and Immunosuppression Withdrawal in Living Donor Liver Transplantation	NCT03164265	2017	University of Pittsburgh Medical Center (US)	Abhinav Humar	No
Safety and Tolerance of Immunomodulating Therapy With Donor-specific Mesenchymal Stem Cells in Pediatric Living-Donor Liver Transplantation, a 24-month, Non-randomized, Open-label, Prospective, Single-center Pilot Trial	NCT02957552	2017	University Children's Hospital (GERMANY)	Ekkehard Sturm	No
Low Dose IL-2 to Expand Endogenous Regulatory T-cells and Achieve Tolerance in Liver Transplantation	NCT02949492<br>2017-000177-37	2017	King's College London (UK)	Alberto Sánchez-Fueyo	No
A Prospective Cohort Study of Operationally Tolerant Allograft Recipients (ITN063ST)	NCT02743793	2016	Immune Tolerance Network (US)	Sindhu Chandran	Yes[c]
Evaluation of Donor Specific Immune Senescence and Exhaustion as Biomarkers of Operational Tolerance Following Liver Transplantation in Adults (ITN056ST)	NCT02533180	2016	Massachusetts General Hospital (US)	James F. Markmann	Yes[d]
Safety of Donor Alloantigen Reactive Tregs to Facilitate Minimization and/or Discontinuation of Immunosuppression in Adult Liver Transplant Recipients (CTOTC-12)	NCT02474199	2016	University of California San Francisco (US)	Sandy Feng	Yes[e]
Prospective randomised marker-based trial to assess the clinical utility and safety of biomarker-guided immunosuppression withdrawal in liver transplantation	2014-004557-14	2015	King's College London (UK)	Alberto Sánchez-Fueyo	No
Liver Immune Tolerance Marker Utilization Study	NCT02541916	2015	Toronto General Hospital (CANADA)	Gary Levy	No

Prospective Randomised Marker-based Trial to Assess the Clinical Utility and Safety of Biomarker-guided Immunosuppression Withdrawal in Liver Transplantation	NCT02498977	2015	King's College Hospital NHS Foundation Trust (UK)	Alberto Sánchez-Fueyo	Yes[f]
Third-party bone marrow-derived mesenchymal stromal cells to induce tolerance in liver transplant recipients	NCT02260375	2014	U A.O. Ospedale Papa Giovanni XXIII (ITALY)	Giuseppe Remuzzi	Yes[g]
Phase 1 Clinical Trial Using Regulatory T Cells as Individualized Medicine to Promote Donor-specific Clinical Liver Transplantation Tolerance in Nanjing	NCT01624077	2014	Nanjing Medical University (PRC)	Ling Lu	No
Pilot Study Evaluating the Safety and Efficacy Profile of Regulatory T Cell Therapy in Liver Transplant Recipients	NCT02166177	2014	King's College Hospital (UK)	Giovanna Lombardi	No
Liver Transplant Tolerance Enhanced By Sirolimus Therapy	NCT02062944	2013	Northwestern University (US)	Josh Levitsky	No
Extracorporeal Photopheresis After Liver Transplant. Phase II Clinical Trial on Safety and Efficacy in Patients With Progressive Withdrawal of Immunosuppression	NCT02090621<br><br>2012-000633-39	2012	Fundación para la Formación e Investigación Sanitaria de la Región de Murcia (SPAIN)	José Antonio Pons	No
Human Umbilical Cord Mesenchymal Stem Cell Induce Liver Allografts Tolerance	NCT01690247	2012	Research Center for Biotherapy (PRC)	Fu-Sheng Wang	No
Immunosuppression Withdrawal for Stable Pediatric Liver Transplant Recipients	NCT01638559	2012	University of California San Francisco (US)	Sandy Feng	Yes[h]
Pilot Study of Immunosuppression Drug Weaning in Liver Recipients Exhibiting Biomarkers of High Likelihood of Tolerance	NCT01445236	2011	Hospital Clinic Barcelona, University of Barcelona (SPAIN)	Alberto Sánchez-Fueyo	No
Gradual Withdrawal of Immunosuppression in Long Term Stable Liver Transplant Recipients Using Immunologic Profile Predicting Operational Tolerance	NCT01198314	2010	Seoul St. Mary's Hospital (SOUTH KOREA)	Jong Young Choi	No
An Observational Study to Assess the Effect of Calcineurin Inhibitors on Markers of Transplant Tolerance	NCT01065584	2010	University Medical Center Goettingen (GERMANY)	Armin Goralczyk	No

Effect of Rapamycin on Tolerance-related Biomarkers on Stable Liver Transplant Recipients	NCT01034345	2009	Hospital Clinic Barcelona, University of Barcelona (SPAIN)	Alberto Sánchez-Fueyo	No
Induction of donor-specific tolerance in patients with liver transplantation with recipient pre-treatment with thymoglobuline and minimal post-transplant immunosuppression	2009-010640-34	2009	Azienda Ospedaliera Policlinico di Modena (ITALY)	-	No
Effect of Immunosuppression Drug Weaning on Hepatitis C Virus Induced Liver Damage After Liver Transplantation	NCT00668369	2008	Hospital Clinic Barcelona, University of Barcelona (SPAIN)	Alberto Sánchez-Fueyo	No
Immune Tolerance and Alloreactivity in Liver Transplant Recipients on Different Monotherapy Immunosuppressive Agents	NCT01678937	2007	Northwestern University (US)	Josh Levitsky	No
Cytokine Kinetics Assay to Assess the Presence or Absence of Tolerance in Organ Transplant Recipients	NCT00585858	2006	University of Wisconsin (US)	-	No
Study of ATEGE-Fresenius Induction in Liver Transplantation Followed by Tacrolimus Weaning	NCT00436722	2006	Hospital Clinic Barcelona, University of Barcelona (SPAIN)	Alberto Sánchez-Fueyo	No
Immunosuppression Withdrawal for Pediatric Living-donor Liver Transplant Recipients (ITN029ST)	NCT00320606	2006	University of California San Francisco (US)	Sandy Feng	Yes[i]
Tacrolimus and pretransplant high-dose r-atg induction vs tacrolimus monotherapy as tolerogenic immunosuppression in adult liver transplantation.an open label, randomised, prospective and controlled trial	2006-004830-34	2006	University Hospitals St Luc (BELGIUM)	-	No
Assessment of the safety and benefit of prospective immunosuppressive drug withdrawal in liver transplantation and prediction of operational tolerance	NCT00647283	2005	Hospital Clinic Barcelona, University of Barcelona (SPAIN)	Alberto Sánchez-Fueyo	No
Thistlethwaite Protocol # ITN025ST - Immunosuppression With Campath-1H and Tacrolimus in Liver Transplantation	NCT00166556	2005	Mayo Clinic (US)	Russell H. Wiesner	No

A Phase II Trial to Assess the Safety of Immunosuppression Withdrawal in Liver Transplant Recipients	NCT00135694	2005	University of Michigan (US)	Abraham Shaked	Yes[j]

[a]Hiroshima University Hospital (JAPAN), Kyoto University Hospital (JAPAN), Nagasaki University Hospital (JAPAN) and Tokyo Women's Medical University Hospital (JAPAN).

[b]HCU Virgen de la Arrixaca (SPAIN), CU de Navarra (SPAIN), Hospital Reina Sofía (SPAIN), Hospital Gregorio Marañón (SPAIN), Hospital Lozano Blesa (SPAIN), CH Universitario A Coruña (SPAIN), Hospital Central de Asturias (SPAIN), H Universitario Ramón y Cajal(SPAIN), CH Universitario de Santiago (SPAIN) and Hospital Río Hortega (SPAIN).

[c]University of California (US), Emory University School of Medicine (US),Ann & Robert H. Lurie Children's Hospital of Chicago (US), University of Pennsylvania Medical Center (US) and Children's Hospital of Pittsburgh of UPMC (US).

[d]University of California, San Francisco Medical Center (US), Northwestern University Feinberg School of Medicine (US), Massachusetts General Hospital (US), Columbia University Medical Center (US), University of Pittsburgh Medical Center (US) and Baylor University Medical Center at Dallas (US).

[e]University of California San Francisco (US), Northwestern University Comprehensive Transplant Ctr (US) and Mayo Clinic in Rochester (US).

[f]King's College Hospital NHS Foundation Trust (UK) and The Newcastle upon Tyne Hospitals NHS Foundation Trust (UK)

[g]AO Ospedale Papa Giovanni XXIII (ITALY), IRCCS-Istituto ki Ricerche Farmacologiche M. Negri (ITALY), Laboratorio G. Lanzani (ITALY) and Policlinico S. Orsola Bologna (ITALY).

[h]University of California San Francisco (US), Children's Hospital of Colorado (US), Emory University and Children's Hospital of Atlanta (US), Ann & Robert H. Lurie Children's Hospital of Chicago (US), University of Michigan C. S. Mott Children's Hospital (US), St. Louis Children's Hospital - Washington University (US), New York Presbyterian Morgan Stanley Children's Hospital - Columbia University Medical Center (US), Cincinnati Children's Hospital (US), Children's Hospital of Philadelphia (US), Texas Children's Hospital (US) and The Hospital for Sick Children (CANADA).

[i]University of California San Francisco (US), Children's Memorial Hospital (US) and Columbia University Medical Center (US).

[j]University of California San Francisco (US), University of Colorado (US), Northwestern University (US), University of Michigan (US), Cleveland Clinic (US), University of Pennsylvania (US), Baylor University (US) and University of Washington (US).

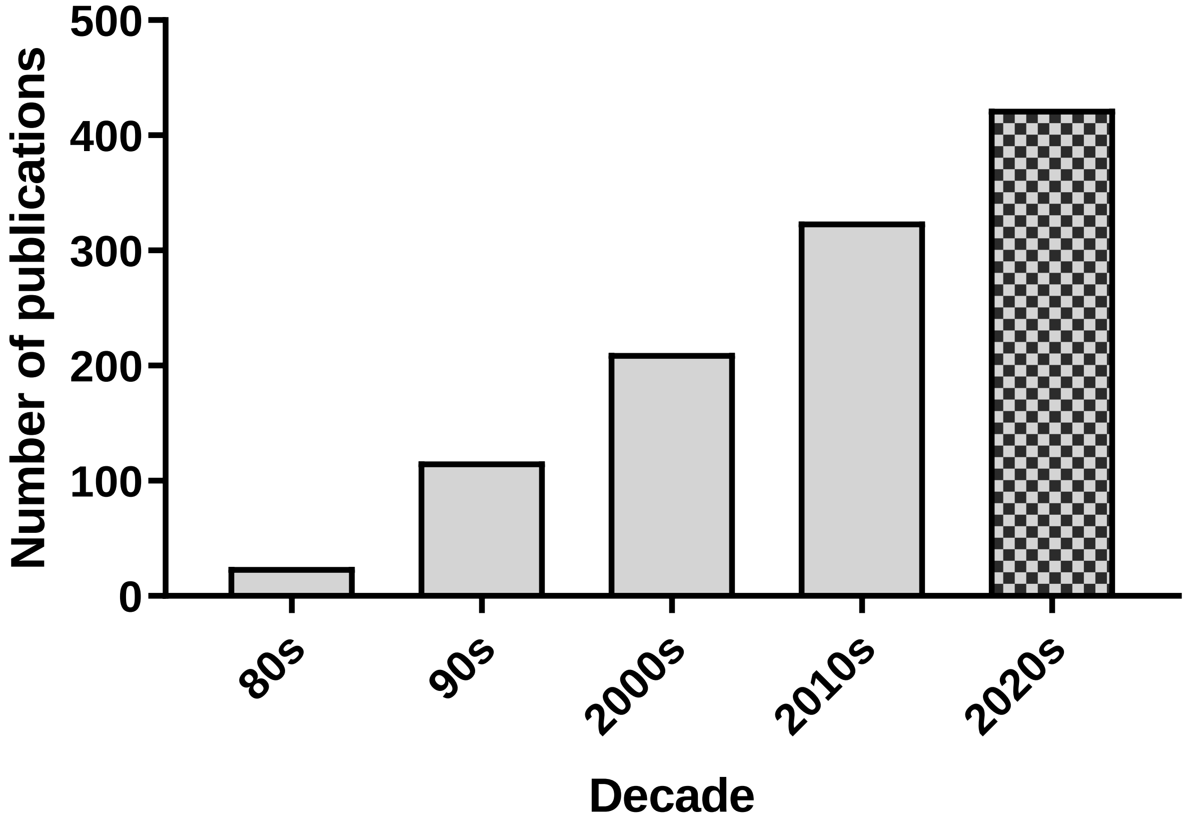

Figure S1.- Number of publications in OT-LT research over time. Graph bar of the number of articles published about OT-LT as separated by decades**.** The publications anticipated for the 2020s decade have been extrapolated from the data collected between 2020 and 2023.

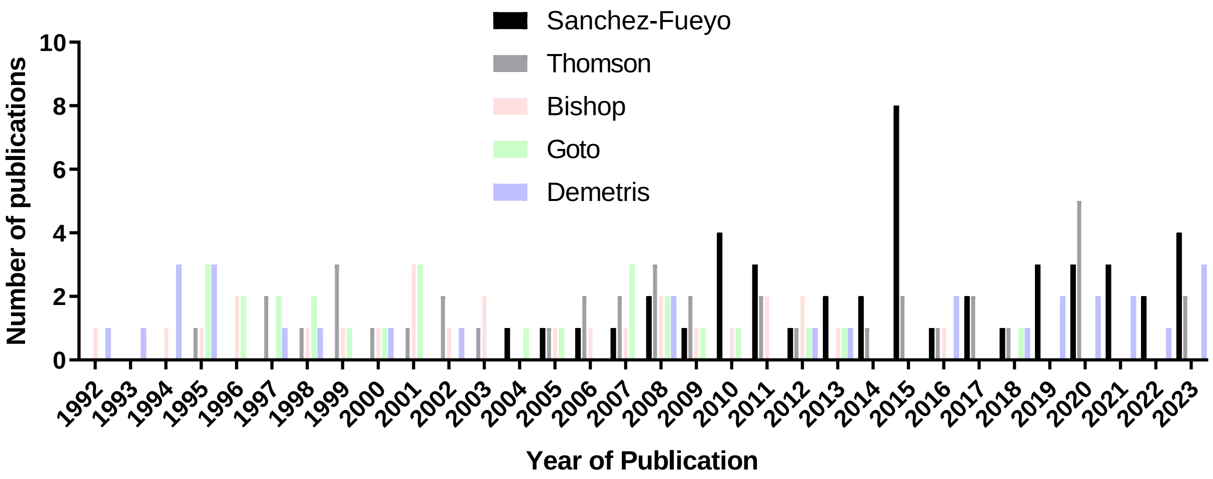

Figure S2.- Number of publications of the five most prolific authors in OT-LT research along the time.

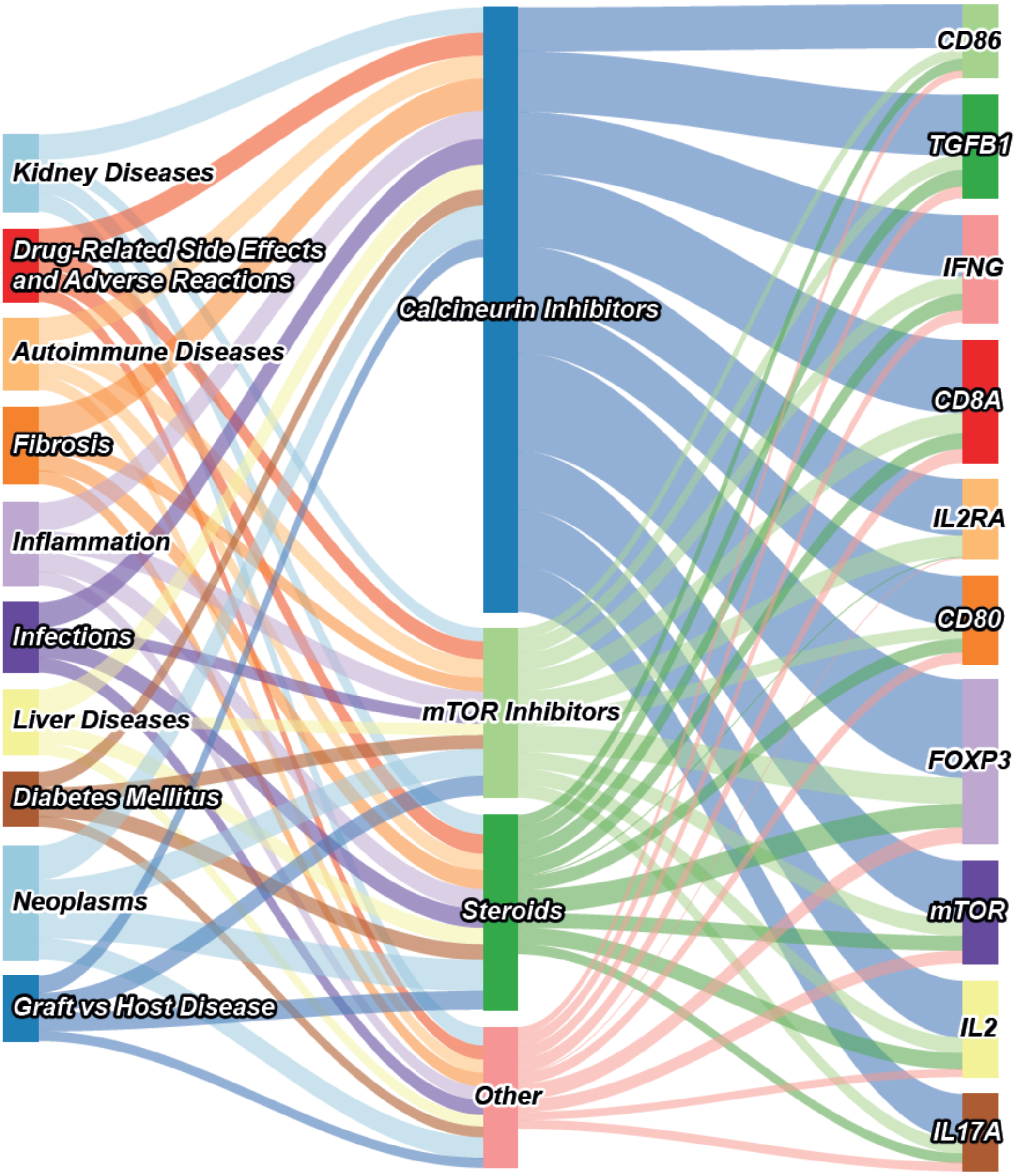

Figure S3.- Keyword co-occurrence in OT-LT research. Sankey diagram of the co-occurrence of the most cited keywords among the three selected clusters (genes, drugs/chemicals and liver transplant related diseases).

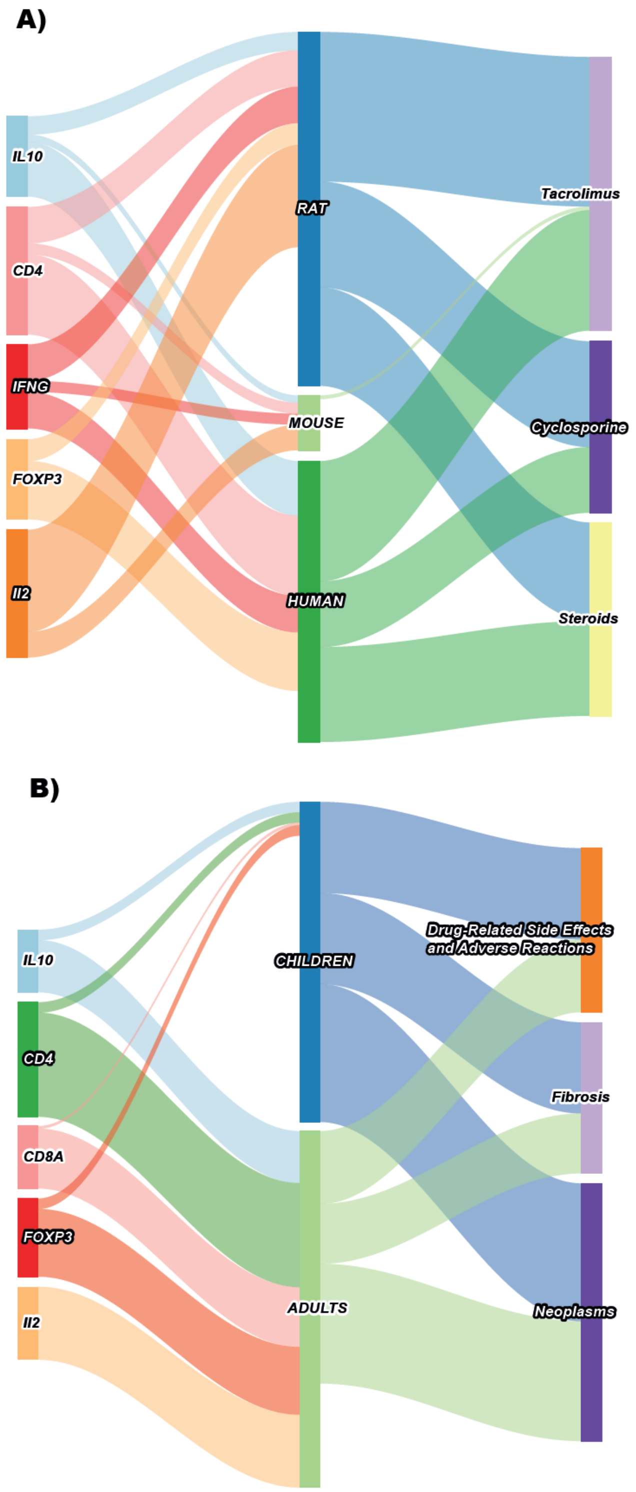

Figure S4.- Keyword analysis by title and abstract. Sankey diagram of the co-occurrence of the most cited keywords among research models (A) and after separating studies on children from those on adults (B).
